# Association of Exposure to Phthalate Metabolites With Sex Hormones, Obesity, and Metabolic Syndrome in US Women

**DOI:** 10.1001/jamanetworkopen.2022.33088

**Published:** 2022-09-23

**Authors:** Pallavi Dubey, Sireesha Y. Reddy, Vishwajeet Singh, Ted Shi, Mallorie Coltharp, Deborah Clegg, Alok K. Dwivedi

**Affiliations:** 1 Department of Obstetrics and Gynecology, Paul L. Foster School of Medicine, Texas Tech University Health Sciences Center, El Paso; 2 Office of Research, Biostatistics and Epidemiology Consulting Lab, Texas Tech University Health Sciences Center, El Paso; 3 Department of Medical Education, Paul L. Foster School of Medicine, Texas Tech University Health Sciences Center, El Paso; 4 Department of Internal Medicine, Paul L. Foster School of Medicine, Texas Tech University Health Sciences Center, El Paso; 5 Division of Biostatistics and Epidemiology, Department of Molecular and Translational Medicine, Paul L. Foster School of Medicine, Texas Tech University Health Sciences Center, El Paso

## Abstract

**Importance:**

Obesity and metabolic syndrome are highly prevalent among the US population and are associated with the dysregulation of sex hormones. An increase in obesity and metabolic syndrome may also be associated with exposure to phthalates. The association of exposure to phthalate metabolites with sex hormones and metabolic health has been understudied in the female population.

**Objective:**

To evaluate the association between exposure to common phthalate metabolites with total testosterone (TT) levels, sex hormone–binding globulin (SHBG) levels, obesity, and metabolic syndrome among women.

**Design, Setting, and Participants:**

This cross-sectional study used data collected from the National Health and Nutrition Examination Survey during 2013 to 2016. Female participants aged 15 years or older with urinary profiles containing common phthalate metabolites were included in this study. Statistical analyses were performed from March 15, 2021, to April 30, 2022.

**Exposures:**

Urinary concentrations of phthalate metabolites were classified into tertiles, and the lowest tertile was used as a reference category. The concentrations of phthalate metabolites and their composite scores based on clustering were also used in the analysis.

**Main Outcomes and Measures:**

Serum concentrations of TT and SHBG were dichotomized into high TT levels (>46 ng/dL [to convert to nanomoles per liter, multiply by 0.0347] for age <50 years and >32 ng/dL for age ≥50 years) and low SHBG levels (<2.85 μg/mL [to convert to nanomoles per liter, multiply by 10.53]) as established for the female population. Obesity was defined as a body mass index of 30 or more (calculated as weight in kilograms divided by height in meters squared), and metabolic syndrome was defined using the National Cholesterol Education Program criteria. The serum concentrations of TT and SHBG were also included in the validation analyses. Modified Poisson models were used to estimate the adjusted relative risk (RR) with 95% CIs for the associations.

**Results:**

Among the 2004 women included in this study, the mean (SD) age was 46.6 (18.5) years (14.7% Hispanic participants, 62.7% non-Hispanic White participants, and 13.2% non-Hispanic Black participants; 17.4% of participants were born outside the US [weighted percentages]; 230 (11.8%) had high TT levels, 210 (10.4%) had low SHBG levels, 825 (39.8%) had obesity, and 965 (45.5%) had metabolic syndrome (weighted percentages). Of the 13 phthalate metabolites, 8 had the highest tertile level greater than 6.2 ng/mL (range, 0.5-75.2 ng/mL). High levels of exposure to mono(2-ethyl-5-carboxypentyl) phthalate (RR, 1.84 [95% CI, 1.33-2.54]), mono(2-ethyl-5-oxohexyl) phthalate (RR, 1.77 [95% CI, 1.21-2.59]), mono(2-ethyl-5-hydroxyhexyl) phthalate (RR, 1.94 [95% CI, 1.34-2.81]), and monobenzyl phthalate (RR, 1.75 [95% CI, 1.21-2.54]) were associated with low SHBG levels but not with high TT levels. High levels of exposure to some of these metabolites were also associated with obesity and metabolic syndrome. Most associations were specific to premenopausal or postmenopausal women.

**Conclusions and Relevance:**

In this cross-sectional study, exposure to certain phthalate metabolites could be associated with low SHBG levels, obesity, and metabolic syndrome depending on menopausal status.

## Key Points

**Question:**

Is exposure to phthalate metabolites associated with levels of sex hormones and metabolic health in premenopausal and postmenopausal women?

**Findings:**

In this cross-sectional study of 2004 US women, exposure to high levels of certain phthalate metabolites was significantly associated with low levels of sex hormone–binding globulin and metabolic health but not associated with total testosterone levels.

**Meaning:**

Findings of this study suggest that there is a potential association of exposures to phthalates with reproductive and metabolic health according to menopausal status.

## Teaser

This cross-sectional study uses data from the National Health and Examination Survey to evaluate the association between exposure to common phthalate metabolites with total testosterone levels, sex hormone-binding globulin levels, obesity, and metabolic syndrome among women.

## Introduction

Obesity and metabolic syndrome are highly prevalent and are considerable risk factors for multiple diseases and mortality in both sexes.^1,2^ Metabolic syndrome and diabetes have rapidly increased in the US, particularly among women.^2,3^ These epidemics are attributed mainly to an interplay between genetic, lifestyle, and environmental factors. Evidence reflects that endocrine-disruptive chemicals (EDCs) may be associated with a marked rise in the incidence of obesity and metabolic syndrome.^4,5^ Endocrine-disruptive chemicals are a cluster of synthetic chemicals that are commonly used in industrial and commercial products and are observed to be pervasive in the environment. Exposure to EDCs can disrupt any action of hormones, resulting in multiple endocrine disorders. Some EDCs, termed obesogens and diabetogens, are causally linked with obesity and diabetes.^5^ Exposure to some EDCs yielded polycystic ovary syndrome–like symptoms among participants in research studies.^6^ Some of the commonly classified EDCs are bisphenol A, phthalates, pesticides, polychlorinated biphenyls, dioxins, and polybrominated biphenyls.^7^ Among EDCs, bisphenol A and phthalates are among the most abundant chemicals in the environment. Although bisphenol A is one of the most studied EDCs in terms of reproductive and metabolic health worldwide, other understudied EDCs, such as phthalates, also have the potential to affect sex hormones and metabolic pathways.^6^

Phthalates are a ubiquitous class of chemicals used as plasticizers in numerous products, including cleaning supplies, medical devices, personal care items and cosmetics, pharmaceuticals, toys, construction, and paints.^8^ However, phthalates do not chemically bind to these products. Humans can also easily be exposed to phthalates owing to direct or indirect contact with these products. Evidence suggests that exposure to phthalates may affect the hypothalamic-pituitary-gonadal axis, total testosterone (TT) levels, and sex hormone–binding globulin (SHBG) levels.^9^ Dysregulation of sex hormones is associated with multiple diseases including endocrine cancers, polycystic ovary syndrome, and metabolic abnormalities. The implications of phthalates for maternal outcomes, sperm concentration and motility, infertility, endometriosis, breast cancer, diabetes, and neurologic disorders have also been identified in a limited number of studies.^10,11,12^ Moreover, exposure to EDCs may induce epigenetic changes, yielding detrimental consequences that can be passed transgenerationally.^6^ Most studies determining the association of phthalate metabolites with sex hormones were based on the male population, pregnant women, or the prepubertal female population,^9,11,13^ with conflicting associations in adult women.^14,15,16^ Although phthalate exposure is widespread and affects all humans, women have 5 to 10 times more exposure to EDCs than men.^17,18^

Phthalates are divided into low molecular weight and high molecular weight (HMW) based on the length of the carbon chain. Low-molecular-weight phthalates are typically used as solvents in personal care products, whereas HMW phthalates are used in polyvinyl chloride plastic products.^19^ Because of exposure to these products in daily routines, phthalates and their metabolites are often detectable in most individuals. Even exposure to low doses of phthalate metabolites may adversely affect human health.^20^ Despite having a detectable amount of phthalate metabolite concentrations in the US population and deleterious consequences that cross generations, the associations of exposures to phthalate metabolites with sex hormones, obesity, and metabolic syndrome have been understudied, particularly among US women. The aim of this study is to examine the association of exposure to phthalate metabolites with sex hormone levels, obesity, and metabolic syndrome. We hypothesize that exposure to certain phthalate metabolites is associated with low SHBG levels, high TT levels, obesity, and metabolic syndrome among women.

## Methods

### Study Population

We analyzed data from the National Health and Nutrition Examination Survey (NHANES), a prospective, ongoing cross-sectional study to examine the health and nutritional status of US individuals. NHANES uses a multistage stratified and cluster survey design to represent noninstitutionalized individuals in the US and is conducted by the National Center for Health Statistics, Centers for Disease Control and Prevention. We used data collected between 2013 and 2016 from NHANES in this study. All reproductive and postmenopausal female individuals aged 15 years or older were included in this study. A total of 7561 individuals were eligible for inclusion after excluding those with extreme TT values. After excluding prepubertal female participants (1410) and individuals with missing phthalate metabolites (4147), a total of 2004 female participants were analyzed. NHANES uses standardized protocols approved by the institutional review board of the Centers for Disease Control and Prevention to collect biological samples for laboratory analyses. The NHANES surveys and examinations obtained written informed consent from all participants after receiving approval from the National Center for Health Statistics Research Ethics Review Board. This cross-sectional study followed the Strengthening the Reporting of Observational Studies in Epidemiology (STROBE) reporting guideline for reporting and analyses.

### Exposure Assessment

Exposure to phthalates was measured through urinary samples from the participants that were collected at the same time as other data and measurements. The detailed protocols for urine sample collection and their analysis, including data processing, quality assessment, and result computation for ensuring high standards, have been described in publications and on the Centers for Disease Control and Prevention website.^14^ A total of 13 metabolites from HMW or low-molecular-weight phthalates were quantified using a high-performance liquid chromatography–electrospray ionization–tandem mass spectrometry method. These metabolites include monocarboxynonyl phthalate (MCNP) of di-isodecyl phthalate (DDP), mono(2-ethyl-5-carboxypentyl) phthalate (MECPP), mono(2-ethylhexyl) phthalate (MEHP), mono(2-ethyl-5-hydroxyhexyl) phthalate (MEHHP), and mono(2-ethyl-5-oxohexyl) phthalate (MEOHP) of di(2-ethylhexyl) phthalate (DEHP); monocarboxyoctyl phthalate (MCOP) and monoisononyl phthalate (MNP) of di-isononyl phthalate (DNP); mono(3-carboxypropyl) phthalate (MCPP) of di-n-octyl phthalate (DiNOP); mono-n-butyl phthalate (MBP) of di-n-butyl phthalate (DBP); monoethyl phthalate (MEP) of di-ethyl phthalate (DEP); mono-isobutyl phthalate (MiBP) of di-isobutyl phthalate; monobenzyl phthalate (MBzP) of benzylbutyl phthalate (BzBP); and cyclohexane-1,2-dicarboxylic acid-mono(hydroxy-isononyl) ester (MHNCH) of 1,2-cyclohexane dicarboxylic acid, di-isononyl ester (DINCH).^21^ We categorized each of the phthalate compound concentrations into tertiles for data analysis, used the lowest tertile as the reference category, and validated the quantitative analysis of metabolite concentrations.

### Sex Hormones Assessment

Serum concentrations of TT were quantified by the liquid chromatography–tandem mass spectrometry method, and serum concentrations of SHBG were quantified by the electrochemiluminescence assay method. We primarily used a TT level greater than 46 ng/dL (to convert to nanomoles per liter, multiply by 0.0347) for age less than 50 years and a TT level greater than 32 ng/dL for age 50 years or older as well as an SHBG level less than 2.85 μg/mL (to convert to nanomoles per liter, multiply by 10.53) as the established and validated cutoff for the female population for assessing androgen excess using the NHANES database.^22^ We also confirmed associations by analyzing TT and SHBG concentrations in the sensitivity analysis.

### Obesity and Metabolic Syndrome Assessments

In NHANES, trained staff record body and laboratory measures at the mobile examination center using standardized methods. The body mass index (BMI; calculated as weight in kilograms divided by height in meters squared) is available for all adults, and we used a BMI of 30 or more to define obesity in this study. As per the National Cholesterol Education Program’s Adult Treatment Panel III, metabolic syndrome is the presence of 3 or more abnormal levels of cardiometabolic symptoms.^23^

### Covariates

Based on previous studies,^14,15,24^ we recorded all potential confounders that included sociodemographic characteristics, such as age (years); race and ethnicity (Hispanic, Non-Hispanic Black, Non-Hispanic White, other [non-Hispanic Asian or other races, including multiracial]); annual household income (<$45 000, $45 000-$99 999, ≥$100 000, or unknown); country of birth (US-born or non–US-born); marital status (married or other [widowed, divorced, separated, never married, living with partner, or unknown]); educational level (≤high school diploma or >high school diploma); health behavior characteristics, including smoking status (yes or no), drinking status (yes or no), and physical activity (low or moderate/vigorous); and laboratory data, including urinary creatinine (milligrams per deciliter). We considered a priori adjustment of all of these potential covariates in multivariable analysis.^25^

### Statistical Analysis

Statistical analysis was performed from March 15, 2021, to April 30, 2022. We used weight-adjusted analyses as appropriate with the NHANES complex survey design in all analyses and followed the statistical reporting guidelines.^23,25^ The adjusted association of each phthalate metabolite with high TT levels, low SHBG levels, obesity, and metabolic syndrome after adjusting for all potential covariates was determined using a survey generalized linear model with a Poisson family distribution and log link.^26^ The overall association of each categorized metabolite with each outcome was also evaluated by a joint test. We further validated the adjusted association of each phthalate with quantitative TT and SHBG levels using survey-weighted linear regression analyses. In the sensitivity analyses, we also evaluated the adjusted association of each phthalate metabolite concentration on the log-transformed scale with log-transformed TT and SHBG levels using weighted multiple linear regression analyses and with obesity and metabolic syndrome using the weighted modified Poisson regression analyses in the overall cohort and by menopausal status. Age 50 years or older was considered postmenopausal, as most studies consider age 50 to 51 years as the reference category for menopause.^27^ The composite exposure of phthalate metabolites was estimated using variable cluster analysis. The multivariable associations of log-transformed values of various groupings of phthalate metabolites with TT levels, SHBG levels, obesity, and metabolic syndrome were examined using survey weight-adjusted linear or Poisson regression analyses. These analyses were also performed according to menopausal status. The final results of generalized linear model analyses were summarized with a relative risk (RR) or a regression coefficient with a 95% CI and *P* value. All statistical analyses were conducted using Stata, version 17 (StataCorp LLC). A 2-tailed *P* < .05 or *P* < .01 after adjusting for multiple comparisons was considered statistically significant. Additional detail related to study methods is included in the eMethods in the Supplement.

## Results

The mean (SD) age of the 2004 female participants was 46.6 (18.5) years, with 14.7% Hispanic participants, 62.7% non-Hispanic White participants, and 13.2% non-Hispanic Black participants; 17.4% of participants were born outside the US (weighted percentages) (Table 1). A total of 230 women (11.8%) had high TT levels (median, 20.2 ng/dL; IQR, 13.6-29.0 ng/dL), 210 (10.4%) had low SHBG levels (median, 567.6 μg/mL; IQR, 379.9-838.9 μg/mL), 825 (39.8%) had obesity (BMI: median, 27.9; IQR, 23.5-33.4), and 965 (45.5%) had metabolic syndrome (weighted percentages). Most of the phthalate metabolites (8 of 13) had the highest tertile level greater than 6.2 (range, 0.5-75.2) ng/mL (eTable 1 in the Supplement). Data-driven clustering among considered phthalate metabolites yielded 4 groups. Groups 1, 2, 3, and 4 were represented by most metabolites of DEHP, DNP, low-molecular-weight phthalates or HMW BzBP, and DINCH, respectively (eTable 2 in the Supplement).

**Table 1.** Participant Characteristics

Characteristic	Participants, No. (%)^a^		
	Overall (N = 2004)	Premenopausal (n = 1135)	Postmenopausal (n = 869)
Age, mean (SD), y	46.6 (18.5)	32.6 (10.0)	64.3 (9.4)
Race and ethnicity			
Hispanic	565 (14.7)	323 (18.7)	242 (9.8)
Non-Hispanic Black	445 (13.2)	261 (14.7)	184 (11.3)
Non-Hispanic White	694 (62.7)	354 (56.2)	340 (71.0)
Other^b^	300 (9.3)	197 (10.5)	103 (7.9)
Educational level			
≤High school diploma	770 (32.1)	325 (26.3)	445 (39.5)
>High school diploma	1015 (61.2)	592 (61.8)	423 (60.4)
Unknown	219 (6.7)	218 (11.9)	1 (0.1)
Marital status			
Married	843 (49.7)	429 (45.1)	414 (55.5)
Other^c^	1160 (50.3)	706 (59.9)	454 (44.5)
Birth country			
US born	1418 (82.6)	808 (80.4)	610 (85.4)
Non-US born	583 (17.4)	326 (19.6)	257 (14.6)
Annual household income, $			
<45 00	1022 (41.4)	556 (41.2)	466 (41.7)
45 000-99 999	506 (28.3)	306 (29.4)	200 (26.9)
≥100 000	313 (23.2)	193 (23.1)	120 (23.3)
Unknown	163 (7.1)	80 (6.3)	83 (8.2)
Smoking status			
No	1269 (61.2)	740 (64.7)	529 (56.6)
Yes	604 (34.8)	265 (28.0)	339 (43.3)
Unknown	131 (4.1)	130 (7.2)	1 (0.1)
Alcohol use status			
No	397 (14.7)	193 (13.4)	204 (16.4)
Yes	1308 (74.0)	691 (70.4)	617 (78.56)
Unknown	299 (11.3)	251 (16.2)	48 (5.1)
Physical activity			
Low	1318 (61.4)	705 (58.5)	613 (65.2)
Moderate or vigorous	685 (38.6)	429 (41.5)	256 (34.9)
Creatinine, mean (SD), mg/dL	0.77 (0.2)	0.72 (0.2)	0.83 (0.3)
High total testosterone level			
No	1774 (88.2)	1019 (89.6)	755 (86.4)
Yes	230 (11.8)	116 (10.5)	114 (13.6)
Total testosterone, median (IQR), ng/dL	20.2 (13.6-29.0)	23.1 (16.5-32.4)	16.3 (11.2-24.4)
Low SHBG level			
No	1657 (89.6)	919 (88.0)	738 (91.7)
Yes	210 (10.4)	130 (12.0)	80 (8.3)
SHBG, median (IQR), μg/mL	6.1 (4.1-9.0)	6.0 (3.9-9.5)	6.1 (4.2-8.6)
Obesity			
No	1162 (60.2)	702 (63.4)	460 (56.2)
Yes	825 (39.8)	422 (36.6)	403 (43.8)
BMI, median (IQR)	27.9 (23.5-33.4)	27.1 (23.0-33.1)	28.8 (24.5-33.5)
Metabolic syndrome			
No	1023 (54.5)	745 (66.7)	278 (39.1)
Yes	965 (45.5)	379 (33.3)	586 (60.9)

Abbreviations: BMI, body mass index (calculated as weight in kilograms divided by height in meters squared); SHBG, sex hormone–binding globulin.

SI conversion factors: To convert creatinine to micromoles per liter, multiply by 88.4; total testosterone to nanomoles per liter, multiply by 0.0347; and SHBG to nanomoles per liter, multiply by 10.53.

^a^ Percentages are weighted.

^b^ Other includes non-Hispanic Asian or other races, including multiracial.

^c^ Other includes widowed, divorced, separated, never married, living with partner, or unknown.

**Figure 1.**
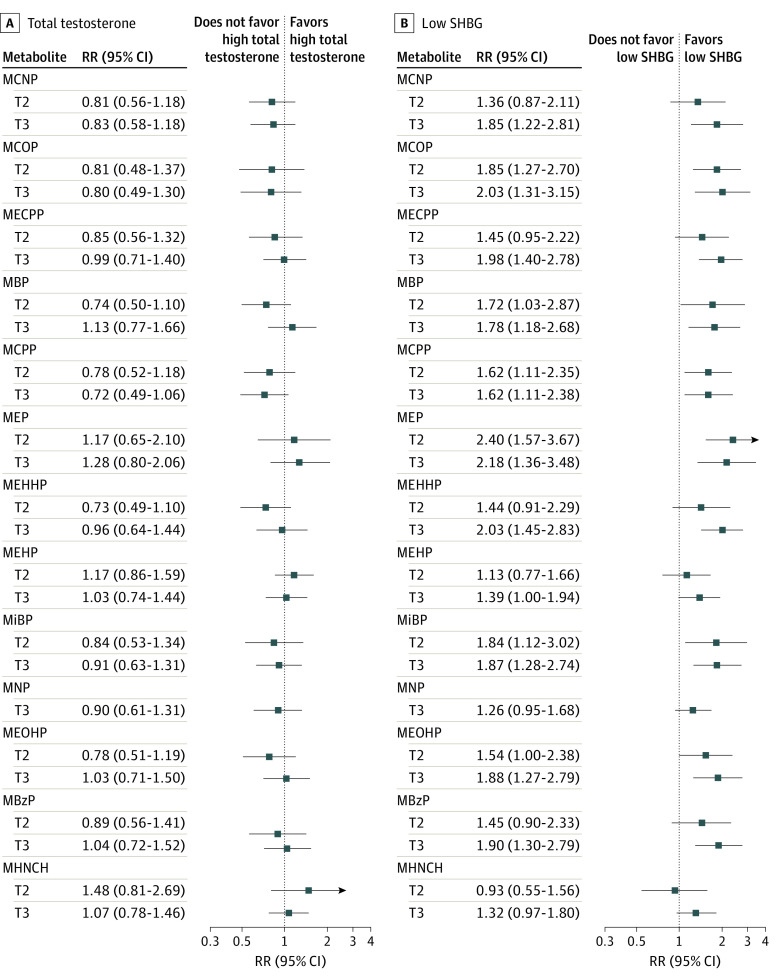
Unadjusted Association of Each Phthalate Metabolite With High Total Testosterone Levels and Low Sex Hormone–Binding Globulin (SHBG) Levels Tertile 1 is the reference category. MBP mono-n-butyl phthalate (ng/mL); MBzP, monobenzyl phthalate (ng/mL); MCNP, monocarboxynonyl phthalate (ng/mL); MCOP, monocarboxyoctyl phthalate (ng/mL); MCPP, mono(3-carboxypropyl) phthalate; MECPP, mono(2-ethyl-5-carboxypenty) phthalate; MEHHP, mono(2-ethyl-5-hydroxyhexyl) phthalate; MEHP, mono(2-ethylhexyl) phthalate (ng/mL); MEOHP, mono(2-ethyl-5-oxohexyl) phthalate; MEP, monoethyl phthalate (ng/mL); MHNCH, cyclohexane-1,2-dicarboxylic acid-mono(hydroxy isononyl) ester (ng/mL); MiBP, mono-isobutyl phthalate; MNP, monoisononyl phthalate (ng/mL); RR, relative risk; T2, second tertile; and T3, third tertile.

None of the metabolites with high exposure were associated with high TT levels in unadjusted (Figure 1) or adjusted analyses (Table 2). However, the middle tertile level of MCOP (regression coefficient, –0.12 [95% CI, –0.22 to –0.02]) was associated with decreased TT concentrations (eTable 3 in the Supplement). Analyses of metabolite concentrations showed that exposure to MHNCH (regression coefficient, 0.06 [95% CI, 0.01-0.11]) was associated with increased TT concentrations only among postmenopausal women (eTable 4 in the Supplement). All phthalate metabolites except MNP and MHNCH were associated with low SHBG levels in unadjusted analysis (Figure 1). In adjusted analysis, low SHBG levels were associated with the middle and high tertiles, respectively, of MCOP (RR, 1.79 [95% CI, 1.19-2.71]; RR, 1.96 [95% CI, 1.21-3.16]), MBP (RR, 1.75 [95% CI, 1.05-2.93]; RR, 1.70 [95% CI, 1.10-2.62]), MCPP (RR, 1.62 [95% CI, 1.09-2.41]; RR, 1.55 [95% CI, 1.04-2.30]), MEP (RR, 2.42 [95% CI, 1.65-3.55]; RR, 2.31 [95% CI, 1.47-3.63]), MiBP (RR, 1.78 [95% CI, 1.09-2.91]; RR, 1.71 [95% CI, 1.15-2.54]), and MEOHP (RR, 1.57 [95% CI, 1.02-2.43]; RR, 1.77 [95% CI, 1.21-2.59]) (Table 2). In addition, the high tertile of MCNP (RR, 1.81 [95% CI, 1.15-2.87]), MECPP (RR, 1.84 [95% CI, 1.33-2.54]), MEHHP (RR, 1.94 [95% CI, 1.34-2.81]), and MBzP (RR, 1.75 [95% CI, 1.21-2.54]) was also associated with low SHBG levels. Some of these associations remained significant even after adjusting for multiple comparisons. The associations of high tertile of phthalate metabolites with lower SHBG concentrations were unchanged in the adjusted analyses except for MBP, MNP, MBzP, MHNCH, and MEOHP (eTable 3 in the Supplement). Most of the metabolite concentrations (MCNP, MCOP, MECPP, MCPP, MiBP, MBzP, and MHNCH) were associated with lower SHBG concentrations to a greater extent among premenopausal women than postmenopausal women (eTable 4 in the Supplement).

**Table 2.** Adjusted Association of Each Phthalate Metabolite With High TT Levels and Low SHBG Levels

Metabolite	High TT level			Low SHBG level		
	RR (95% CI)^a^	*P* value, overall	*P* value	RR (95% CI)^a^	*P* value, overall	*P* value
**MCNP**						
T1	1 [Reference]	.44	NA	1 [Reference]	.05	NA
T2	0.80 (0.53-1.20)		.26	1.35 (0.84-2.16)		.21
T3	0.82 (0.56-1.18)		.27	1.81 (1.15-2.87)		.01^b^
**MCOP**						
T1	1 [Reference]	.66	NA	1 [Reference]	.02	NA
T2	0.80 (0.47-1.35)		.39	1.79 (1.19-2.71)		.007^b^
T3	0.80 (0.48-1.34)		.39	1.96 (1.21-3.16)		.008^b^
**MECPP**						
T1	1 [Reference]	.68	NA	1 [Reference]	.003^b^	NA
T2	0.85 (0.55-1.30)		.44	1.43 (0.94-2.16)		.09
T3	1.02 (0.72-1.45)		.91	1.84 (1.33-2.54)		.001^b^
**MBP**						
T1	1 [Reference]	.08	NA	1 [Reference]	.04	NA
T2	0.77 (0.53-1.12)		.17	1.75 (1.05-2.93)		.03
T3	1.09 (0.74-1.62)		.64	1.70 (1.10-2.62)		.02
**MCPP**						
T1	1 [Reference]	.20	NA	1 [Reference]	.03	NA
T2	0.73 (0.49-1.10)		.12	1.62 (1.09-2.41)		.02
T3	0.72 (0.48-1.07)		.10	1.55 (1.04-2.30)		.03
**MEP**						
T1	1 [Reference]	.69	NA	1 [Reference]	<.001^b^	NA
T2	1.17 (0.66-2.09)		.57	2.42 (1.65-3.55)		<.001^b^
T3	1.24 (0.75-2.03)		.39	2.31 (1.47-3.63)		.001^b^
**MEHHP**						
T1	1 [Reference]	.26	NA	1 [Reference]	.003^b^	NA
T2	0.71 (0.46-1.08)		.11	1.45 (0.92-2.29)		.10
T3	0.96 (0.64-1.44)		.84	1.94 (1.34-2.81)		.001^b^
**MEHP**						
T1	1 [Reference]	.44	NA	1 [Reference]	.45	NA
T2	1.20 (0.88-1.64)		.24	1.11 (0.75-1.64)		.60
T3	1.01 (0.71-1.43)		.95	1.23 (0.86-1.77)		.24
**MiBP**						
T1	1 [Reference]	.60	NA	1 [Reference]	.02	NA
T2	0.78 (0.48-1.28)		.31	1.78 (1.09-2.91)		.02
T3	0.88 (0.61-1.25)		.45	1.71 (1.15-2.54)		.01^b^
**MNP**						
T1/T2	1 [Reference]	.54	NA	1 [Reference]	.42	NA
T3	0.89 (0.61-1.30)		.54	1.12 (0.85-1.48)		.42
**MEOHP**						
T1	1 [Reference]	.31	NA	1 [Reference]	.02	NA
T2	0.76 (0.49-1.17)		.20	1.57 (1.02-2.43)		.04
T3	1.05 (0.72-1.55)		.78	1.77 (1.21-2.59)		.005^b^
**MBzP**						
T1	1 [Reference]	.82	NA	1 [Reference]	.02	NA
T2	0.87 (0.55-1.37)		.53	1.44 (0.90-2.29)		.13
T3	0.94 (0.64-1.37)		.72	1.75 (1.21-2.54)		.004^b^
**MHNCH**						
T1	1 [Reference]	.51	NA	1 [Reference]	.57	NA
T2	1.41 (0.78-2.54)		.25	0.86 (0.51-1.48)		.58
T3	0.98 (0.72-1.34)		.89	1.18 (0.84-1.67)		.34

Abbreviations: MBP, mono-n-butyl phthalate (ng/mL); MBzP, monobenzyl phthalate (ng/mL); MCNP, monocarboxynonyl phthalate (ng/mL); MCOP, monocarboxyoctyl phthalate (ng/mL); MCPP, mono(3-carboxypropyl) phthalate; MECPP, mono(2-ethyl-5-carboxypenty) phthalate; MEHHP, mono(2-ethyl-5-hydroxyhexyl) phthalate; MEHP, mono(2-ethylhexyl) phthalate (ng/mL); MEOHP, mono(2-ethyl-5-oxohexyl) phthalate; MEP, monoethyl phthalate (ng/mL); MHNCH, cyclohexane-1,2-dicarboxylic acid-mono(hydroxy isononyl) ester (ng/mL); MiBP, mono-isobutyl phthalate; MNP, monoisononyl phthalate (ng/mL); NA, not applicable; RR, relative risk; SHBG, sex hormone–binding globulin; T1, first tertile (reference category); T2, second tertile; T3, third tertile; TT, total testosterone.

^a^ All analyses adjusted for age, race and ethnicity, income, marital status, birth country, educational level, smoking status, alcohol use status, physical activity, and creatinine level.

^b^ *P* values are significant at a 1% level of significance after adjusting for multiple comparisons. The overall *P* value provides an overall association between each phthalate metabolite and each outcome, whereas the *P* value provides the association of each level of phthalate metabolite with each outcome.

**Figure 2.**
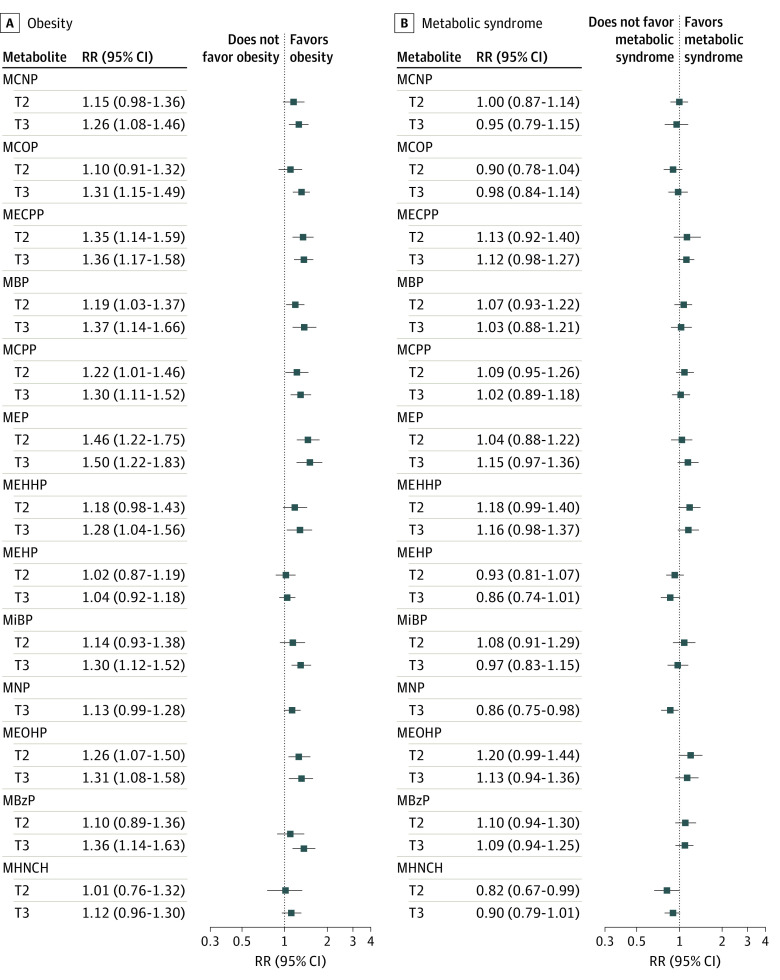
Unadjusted Association of Each Phthalate Metabolite With Obesity and Metabolic Syndrome Tertile 1 is the reference category. MBP mono-n-butyl phthalate (ng/mL); MBzP, monobenzyl phthalate (ng/mL); MCNP, monocarboxynonyl phthalate (ng/mL); MCOP, monocarboxyoctyl phthalate (ng/mL); MCPP, mono(3-carboxypropyl) phthalate; MECPP, mono(2-ethyl-5-carboxypenty) phthalate; MEHHP, mono(2-ethyl-5-hydroxyhexyl) phthalate; MEHP, mono(2-ethylhexyl) phthalate (ng/mL); MEOHP, mono(2-ethyl-5-oxohexyl) phthalate; MEP, monoethyl phthalate (ng/mL); MHNCH, cyclohexane-1,2-dicarboxylic acid-mono(hydroxy isononyl) ester (ng/mL); MiBP, mono-isobutyl phthalate; MNP, monoisononyl phthalate (ng/mL); RR, relative risk; T2, second tertile; and T3, third tertile.

All metabolites of phthalates except MNP and MHNCH were associated with obesity in the unadjusted analysis (Figure 2), and all metabolites of phthalates except MEHP and MHNCH were also associated with obesity in the adjusted analysis (Table 3). Both moderate and high tertiles, respectively, of MECPP (RR, 1.33 [95% CI, 1.13-1.56]; RR, 1.36 [95% CI, 1.17-1.58]), MBP (RR, 1.17 [95% CI, 1.01-1.36]; RR, 1.37 [95% CI, 1.12-1.66]), MEP (RR, 1.45 [95% CI, 1.20-1.76]; RR, 1.39 [95% CI, 1.13-1.73]), and MEOHP (RR, 1.24 [95% CI, 1.05-1.47]; RR, 1.30 [95% CI, 1.08-1.57]) were associated with obesity. Most of the phthalate metabolites were associated with obesity even after adjusting for multiple comparisons. Some metabolite concentrations (MBP, MiBP, MEOHP, MBzP, and MHNCH) were associated with obesity among premenopausal women to a greater extent than among postmenopausal women. Similarly, some metabolite concentrations (MCNP, MCOP, MECPP, MCPP, and MEP) were associated with obesity among postmenopausal women to a greater extent than among premenopausal women (eTable 5 in the Supplement). None of the metabolites except MNP were associated with metabolic syndrome in unadjusted analysis (Figure 2). Only high levels of MECPP (RR, 1.16 [95% CI, 1.02-1.32]), MEHHP (RR, 1.21 [95% CI, 1.02-1.44]), MBzP (RR, 1.19 [95% CI, 1.02-1.38]), and MEOHP (RR, 1.20 [95% CI, 1.00-1.43]) were associated with an increased prevalence of metabolic syndrome (Table 3). However, these associations were not significant after adjusting for multiple comparisons. The concentrations of MECPP, MEHPP, MEOHP, and MHNCH metabolites were associated with metabolic syndrome among premenopausal women, whereas increasing MBzP concentrations were associated with metabolic syndrome among postmenopausal women (eTable 5 in the Supplement).

**Table 3.** Adjusted Association of Each Phthalate Metabolite With Obesity and Metabolic Syndrome

Metabolite	Obesity			Metabolic syndrome		
	RR (95% CI)^a^	*P* value, overall	*P* value	RR (95% CI)^a^	*P* value, overall	*P* value
**MCNP**						
T1	1 [Reference]	.07	NA	1 [Reference]	.87	NA
T2	1.10 (0.94-1.29)		.21	0.98 (0.86-1.12)		.73
T3	1.21 (1.03-1.42)		.02	1.01 (0.85-1.19)		.94
**MCOP**						
T1	1 [Reference]	<.001^b^	NA	1 [Reference]	.26	NA
T2	1.07 (0.88-1.29)		.48	0.93 (0.80-1.08)		.33
T3	1.29 (1.13-1.48)		<.001^b^	1.05 (0.91-1.22)		.50
**MECPP**						
T1	1 [Reference]	<.001^b^	NA	1 [Reference]	.05	NA
T2	1.33 (1.13-1.56)		.001^b^	1.10 (0.90-1.35)		.33
T3	1.36 (1.17-1.58)		<.001^b^	1.16 (1.02-1.32)		.02
**MBP**						
T1	1 [Reference]	.01^b^	NA	1 [Reference]	.70	NA
T2	1.17 (1.01-1.36)		.03	1.06 (0.92-1.22)		.42
T3	1.37 (1.12-1.66)		.003^b^	1.06 (0.90-1.25)		.46
**MCPP**						
T1	1 [Reference]	.01^b^	NA	1 [Reference]	.60	NA
T2	1.17 (0.97-1.42)		.11	1.05 (0.90-1.22)		.53
T3	1.29 (1.09-1.51)		.003^b^	1.07 (0.94-1.21)		.32
**MEP**						
T1	1 [Reference]	.001^b^	NA	1 [Reference]	.58	NA
T2	1.45 (1.20-1.76)		<.001^b^	1.07 (0.91-1.25)		.42
T3	1.39 (1.13-1.73)		.003^b^	1.09 (0.92-1.30)		.30
**MEHHP**						
T1	1 [Reference]	.09	NA	1 [Reference]	.09	NA
T2	1.15 (0.95-1.40)		.15	1.14 (0.96-1.36)		.12
T3	1.26 (1.03-1.54)		.03	1.21 (1.02-1.44)		.03
**MEHP**						
T1	1 [Reference]	.70	NA	1 [Reference]	.83	NA
T2	1.03 (0.89-1.19)		.65	0.96 (0.85-1.09)		.54
T3	1.05 (0.93-1.19)		.44	0.98 (0.84-1.15)		.81
**MiBP**						
T1	1 [Reference]	.01^b^	NA	1 [Reference]	.65	NA
T2	1.10 (0.91-1.33)		.31	1.07 (0.90-1.28)		.40
T3	1.29 (1.10-1.51)		.003^b^	1.07 (0.91-1.27)		.40
**MNP**						
T1/T2	1 [Reference]	.04	NA	1 [Reference]	.93	NA
T3	1.16 (1.01-1.33)		.04	1.01 (0.89-1.13)		.93
**MEOHP**						
T1	1 [Reference]	.02	NA	1 [Reference]	.14	NA
T2	1.24 (1.05-1.47)		.01^b^	1.17 (0.98-1.39)		.08
T3	1.30 (1.08-1.57)		.007^b^	1.20 (1.00-1.43)		.05
**MBzP**						
T1	1 [Reference]	.005^b^	NA	1 [Reference]	.07	NA
T2	1.08 (0.87-1.33)		.48	1.12 (0.95-1.32)		.17
T3	1.34 (1.10-1.62)		.004^b^	1.19 (1.02-1.38)		.03
**MHNCH**						
T1	1 [Reference]	.29	NA	1 [Reference]	.55	NA
T2	1.03 (0.78-1.36)		.84	0.91 (0.75-1.10)		.32
T3	1.13 (0.96-1.33)		.13	1.03 (0.92-1.15)		.61

Abbreviations: MBP, mono-n-butyl phthalate (ng/mL); MBzP, monobenzyl phthalate (ng/mL); MCNP, monocarboxynonyl phthalate (ng/mL); MCOP, monocarboxyoctyl phthalate (ng/mL); MCPP, mono(3-carboxypropyl) phthalate; MECPP, mono(2-ethyl-5-carboxypenty) phthalate; MEHHP, mono(2-ethyl-5-hydroxyhexyl) phthalate; MEHP, mono(2-ethylhexyl) phthalate (ng/mL); MEOHP, mono(2-ethyl-5-oxohexyl) phthalate; MEP, monoethyl phthalate (ng/mL); MHNCH, cyclohexane-1,2-dicarboxylic acid-mono(hydroxy isononyl) ester (ng/mL); MiBP, mono-isobutyl phthalate; MNP, monoisononyl phthalate (ng/mL); NA, not applicable; RR, relative risk; T1, first tertile (reference category); T2, second tertile; T3, third tertile.

^a^ All analyses adjusted for age, race and ethnicity, income, marital status, birth country, educational level, smoking status, alcohol use status, physical activity, and creatinine level.

^b^ *P* values are significant at a 1% level of significance after adjusting for multiple comparisons. The overall *P* value provides an overall association between each phthalate metabolite and each outcome, whereas the *P* value provides the association of each level of phthalate metabolite with each outcome.

In the adjusted analyses of combined phthalate metabolites, the composite scores of HMW metabolites or some groupings of HMW metabolites were associated with low SHBG concentrations (regression coefficient, –0.09 [95% CI, –0.13 to –0.05]) and obesity (RR, 1.12 [95% CI, 1.04-1.21]) (eTables 6 and 7 and eAppendix in the Supplement). The associations of composite scores of HMW metabolites with lower SHBG concentrations were more pronounced among premenopausal women than postmenopausal women (eTable 6 and eAppendix in the Supplement). However, the associations of composite scores of HMW metabolites with obesity were dependent on menopausal status (eTable 7 and eAppendix in the Supplement).

## Discussion

Consistent with our hypothesis, our findings suggest that high levels of exposure to some phthalate metabolites are associated with lower SHBG levels, obesity, and metabolic syndrome to a greater extent among premenopausal women than among postmenopausal women. However, some phthalate metabolites were more markedly associated with obesity among postmenopausal women. Decreasing SHBG concentrations have been associated with multiple diseases including obesity, metabolic abnormalities, and polycystic ovary syndrome, as well as hormone-sensitive cancers in the female population.^22^ A computational study demonstrated a commonality between phthalate compounds and SHBG levels, suggesting that exposure to phthalates has the potential to disrupt the endocrine and reproductive functions of SHBG.^28^ One study found lower SHBG concentrations among pregnant women using hair products and cosmetics compared with women not using hair products and cosmetics.^29^ Because phthalates are commonly used in personal care and cosmetic products, a study investigated the use of 18 phthalates in personal care and cosmetic products across Canada.^30^ The authors observed that DEP was largely detected in most products, with the highest daily exposure among women. Similarly, we also observed the highest concentration of the MEP metabolite of DEP in our study. In our study, a high exposure to the MEP metabolite of DEP was one of the most prominent factors for low SHBG levels and obesity. Monoethyl phthalate was inversely associated with SHBG levels during pregnancy,^31^ similar to findings in our study of premenopausal and postmenopausal women.

Although most high levels of phthalate metabolites were associated with low SHBG levels in our study, the composite exposure of HMW phthalates, especially metabolites of DNP, DiNOP, DDP, BzBP, and DINCH, was associated with decreasing SHBG concentrations. Confirming our findings, the long-chain phthalates, such as DiNOP, DNP, and DINCH, have the highest binding affinity to the SHBG ligand, suggesting that metabolites of these phthalates can have detrimental consequences for hormone homeostatic functions.^28,32^ In female animals, DBP and BzBP metabolites have been shown to induce ovarian abnormalities.^33^ Like phthalate metabolites, the other EDCs, such as paraben concentrations, were also found to be inversely associated with SHBG concentrations.^34^ Unlike our study, a study by Chiang et al^24^ did not find an association between SHBG concentrations and metabolite concentrations. However, the authors reported an inverse association between SHBG concentrations and MCPP among women with overweight. Contrary to our study findings, Zhu et al^14^ used the NHANES database and reported no association between SHBG concentrations and exposure to phthalate metabolites among women. This finding could be because these authors did not account for the weighting structure of complex survey data and collinearity among phthalate metabolites. In addition, our study differs from the study by Zhu et al^14^ in the following aspects: NHANES study years, study samples, outcomes, and data analysis, including the methods and adjustments of confounders.

Exposure to phthalate metabolites was consistently associated with obesity in our study. Multiple studies have reported positive associations between urinary phthalate concentrations and increased waist circumference among the male and female populations.^35,36^ One study evaluated the associations of MBzP and MEHP with BMI using NHANES cycles of 1999-2002 data sets.^37^ That study found that MEHP was associated with increased BMI and waist circumference among female individuals aged between 12 and 59 years. Similarly, another NHANES-based study evaluated associations between DEHP metabolites and BMI and cardiometabolic markers.^38^ The authors found that only MBP and MEHP were positively associated with BMI, without any association with other metabolic abnormalities. In contrast, we found that high tertiles (higher concentrations) of phthalate metabolites, such as MECPP, MEOHP, MEHHP, and MBzP, were associated with metabolic syndrome. Studies have reported conflicting associations of phthalates with metabolic syndrome and diabetes. In some studies, these associations were found in male participants but not in female participants.^8,12,38^ Other studies supported our findings, demonstrating that some phthalate compounds, such as MBzP and MiBP, were associated with cardiometabolic risk, including diabetes, in female participants.^3,39^ These conflicting associations mostly occurred owing to differences in the age groups, statistical methods, and consideration of confounders in data analyses.

Consistent with one study,^15^ our study did not find an association between high TT levels and phthalate metabolites. Contrary to our study, some studies have reported an inverse association between phthalate exposure and TT levels.^14,16^ However, most studies depicting an inverse association between phthalate metabolites and TT levels were conducted among men.^14,40,41^ Our study identified an association between MHNCH and higher TT concentrations only among postmenopausal women. Long et al^15^ also reported a positive association between DINCH and bioavailable testosterone, free testosterone, and free androgen index among postmenopausal women, indicating a role of the MHNCH metabolite of DINCH among postmenopausal women. We observed associations of some metabolite concentrations with SHBG levels, obesity, and metabolic syndrome among premenopausal women to a greater extent than among postmenopausal women, similar to associations found by Chiang et al.^24^ A study by James-Todd et al^3^ also reported associations of higher quartiles of MBzP and DEHP with metabolic syndrome among premenopausal women only. This finding may be because of increased exposure to phthalates at earlier ages compared with later ages, mainly owing to increased use of personal care products. Furthermore, the hypothalamic-pituitary-gonadal axis and ovaries may be less affected by phthalate-induced alterations during the menopausal transition.^24^ Our clustering analysis yielded reasonable clusters, as most of the metabolites of the parent phthalates were obtained from the same group. However, some individual or combined exposures to phthalate metabolites were associated with obesity depending on menopausal status. This finding might be owing to differences in the expression of estrogen receptors between premenopausal and postmenopausal women. Phthalates may be associated with estrogen levels and age at menopause, contributing to different associations with obesity by menopause status.^42,43^ It is postulated that exposure to EDCs can have a more implications for clinical outcomes among menopausal women.^15^

Although the mechanism of phthalates’ association with sex hormones and metabolic abnormalities is not fully understood, accumulating epidemiological evidence suggests that higher exposure to phthalates may be associated with steroidogenesis and SHBG function by disrupting the hypothalamic-pituitary-gonadal axis and associated sex hormone action.^11^ In addition, phthalates may directly bind to SHBG, affecting sex hormone function in the body.^32^ The association of phthalates with obesity could be owing to interference with adipose tissue function by disrupting the peroxisome proliferator–activated receptors (PPAR-γ) or PPAR-α or disrupting energy balance or endocrine steroid homeostasis.^44,45^ The association of phthalates with metabolic syndrome could be owing to increasing obesity by binding to PPAR-γ or PPAR-α or altering endocrine hormone function or owing to altered β-cell function.^3,45,46^ Based on multiple reports and our data, it is likely that metabolic abnormalities, including obesity, are associated with exposure to phthalates via the SHBG pathway in the female population.^8^

### Strengths and Limitations

Our study has some strengths. We conducted this study by analyzing the most recent data reporting phthalate compounds and sex hormones from a well-established, population-based NHANES database. We comprehensively included understudied phthalate metabolites in women of reproductive age and postmenopausal women, and we included most critical covariates in our adjusted analyses. We validated our findings by performing numerous sensitivity analyses, including categorized and quantitative metabolite concentrations incorporating the weighting structure of data.

This study also has some limitations. Because of a cross-sectional design, we were unable to establish a causal link between phthalate metabolites and sex hormones and metabolic abnormalities. Although we have adjusted all of the critical factors in this study, there may be possibilities of unaccounted or unobserved confounders, such as medical conditions, diet, occupational status, timing of the sample collection, medication use, substance use, and type and frequency of use of phthalate-containing products, in the analysis that may produce biased associations. However, adjustments for some of these factors did not alter associations in other studies.^3,15,47,48^ Furthermore, the complex interactions across metabolites may persist and require further evaluation. The levels of phthalate exposure and their timing of metabolism into different metabolites need to be assessed to establish the relative toxic effects of all phthalates, including their hydrolyzed and conjugated products, in future studies. Because of the lack of standardized thresholds of phthalate metabolites, a direct comparison of effect size across studies may not be feasible. However, the cutoff of high-risk phthalate metabolites may be standardized using our analysis of tertiles of phthalate metabolites for comparative evaluation and developing feasible strategies to minimize exposure to these compounds. Moreover, the study cannot distinguish the association of short-term or long-term exposure to phthalates with outcomes. Although we analyzed the most updated NHANES data, data collection for phthalate profiling of only one-third of the survey participants may affect the generalizability of our study findings.

## Conclusions

This cross-sectional study found that exposure to most phthalate metabolites is associated with low SHBG levels and obesity to a greater extent among premenopausal women than postmenopausal women. However, individual or composite exposure to HMW phthalates, especially from DEHP, DNP, DINCH, and BzBP, may have differential associations with SHBG levels, obesity, and metabolic abnormalities according to menopausal status. Our findings indicate that screening of some phthalates may be critical for women with obesity, particularly those with low SHBG levels. Community and public awareness is required to avoid the use of products containing harmful plasticizers and substances. In addition, in the future, authorities may develop feasible strategies and impose regulations and restrictions for the use of harmful and toxic phthalates in their products. Future longitudinal and mechanistic studies are required to confirm our findings and explore possible sources of early intervention to preserve the reproductive and metabolic health of US adult women.

## Notes

Supplement.
eMethods.
**eAppendix. **Supplemental Results **eTable 1.** Distribution of Exposure to Phthalate Metabolites **eTable 2.** Cluster Analysis of Metabolites of Phthalates **eTable 3.** Adjusted Association of Each Phthalate Metabolite With TT and SHBG Concentrations **eTable 4.** Adjusted Association of Each Phthalate Metabolite Concentration With TT and SHBG Concentrations **eTable 5.** Adjusted Association of Each Phthalate Metabolite Concentration With Obesity and Metabolic Syndrome **eTable 6.** Adjusted Associations of Composite Scores of Phthalate Metabolites With TT and SHBG Concentrations **eTable 7.** Adjusted Associations of Composite Scores of Phthalate Metabolites With Obesity and Metabolic Syndrome
