# Supplementary material for: Association of Exposure to Phthalate Metabolites With Sex Hormones, Obesity, and Metabolic Syndrome in US Women
Source: JAMA Netw Open. 2022 Sep 23;5(9):e2233088. doi: 10.1001/jamanetworkopen.2022.33088 (PMC9508659; doi:10.1001/jamanetworkopen.2022.33088)
Supplement: Supplement. — eMethods. eAppendix. Supplemental Results eTable 1. Distribution of Exposure to Phthalate Metabolites eTable 2. Cluster Analysis of Metabolites of Phthalates eTable 3. Adjusted Association of Each Phthalate Metabolite With TT and SHBG Concentrations eTable 4. Adjusted Association of Each Phthalate Metabolite Concentration With TT and SHBG Concentrations eTable 5. Adjusted Association of Each Phthalate Metabolite Concentration With Obesity and Metabolic Syndrome eTable 6. Adjusted Associations of Composite Scores of Phthalate Metabolites With TT and SHBG Concentrations eTable 7. Adjusted Associations of Composite Scores of Phthalate Metabolites With Obesity and Metabolic Syndrome [file jamanetwopen-e2233088-s001.pdf]

Supplementary Online Content

Dubey P, Reddy SY, Singh V, et al. Association of exposure to phthalate metabolites with sex hormones, obesity, and metabolic syndrome in US women. *JAMA Netw Open.* 2022;5(9):e2233088. doi:10.1001/jamanetworkopen.2022.33088

eMethods.
eAppendix. Supplemental Results
eTable 1. Distribution of Exposure to Phthalate Metabolites
eTable 2. Cluster Analysis of Metabolites of Phthalates
eTable 3. Adjusted Association of Each Phthalate Metabolite With TT and SHBG Concentrations
eTable 4. Adjusted Association of Each Phthalate Metabolite Concentration With TT and SHBG Concentrations
eTable 5. Adjusted Association of Each Phthalate Metabolite Concentration With Obesity and Metabolic Syndrome
eTable 6. Adjusted Associations of Composite Scores of Phthalate Metabolites With TT and SHBG Concentrations
eTable 7. Adjusted Associations of Composite Scores of Phthalate Metabolites With Obesity and Metabolic Syndrome

This supplementary material has been provided by the authors to give readers additional information about their work.

© 2022 Dubey P et al. *JAMA Network Open.*

eMethods.

Study population: The two cycles included in this study provide comprehensive data on exposures and outcomes considered in this study. During these two cycles, the NHANES oversampled some individuals to better represent all ethnic groups. The NHANES utilizes standardized protocols approved by the institutional review board of the CDC to collect biological samples for laboratory analyses. All the sample collections and examinations were conducted by highly trained personnel. Since NHANES collects only a one-third subsample of all participants for phthalates analyses (https://wwwn.cdc.gov/Nchs/Nhanes/2013-2014/PHTHTE_H.htm), it is reasonable to have a large proportion of missing metabolite data.

Exposure assessment: Although different studies might have used different abbreviations for the metabolites, we followed the acronyms provided by the NHANES documentation (https://wwwn.cdc.gov/Nchs/Nhanes/2013-2014/PHTHTE_H.htm). The NHANES utilizes isotopically-labeled internal standards to increase assay precision and accuracy for measuring concentrations of metabolites in the low ng/mL range from urine samples. A very minimum number of samples (<5%) had concentrations below the limits of detection (LOD) except for MHNC and MCPP. The range of LOD of different metabolites was 0.2 to 1.2 ng/mL and instrument reading was used for concentrations below the LODs. The metabolite concentrations were categorized into tertiles to obtain sufficient number of outcomes in each category.

Outcome assessment: The NHANES implements quality assurance and control protocols for ensuring high accuracy and precision. Serum samples were collected, appropriately stored, and analyzed using the standard protocols for measuring androgen concentrations. Details of sample processing, method performance, quality control assessment, and analytical and result interpretation are reported by the CDC. The LOD was 0.7475 for TT and 0.800 nmol/L for SHBG.

© 2022 Dubey P et al. *JAMA Network Open.*

As per the National Cholesterol Education Program (NCEP)'s Adult Treatment Panel III, MS is the presence of three or more abnormal levels of cardiometabolic symptoms including glucose (FG) or hemoglobin A1c (HbA1c), waist circumference (WC), triglyceride (TG), high-density lipoprotein (HDL) cholesterol, and systolic and diastolic blood pressures (SBP and DBP). We defined MS using the NCEP criteria and considered MS if individuals were present with at least 3 symptoms of the following: FG level at least 100 mg/dL or HbA1c> 6 or taking diabetes medications, WC> 88 cm, TG> 150 mg/dL, HDL<50 mg/dL, and SBP ≥130 or DBP ≥85 or taking hypertension medications.

Statistical analysis: We described the distribution of metabolite concentrations using centiles. Mean with standard deviation (SD) or median with interquartile range (IQR) was used to describe continuous outcome data while categorical variables were summarized with proportions. Based on previous studies and clinical applications[22], the primary analyses were carried out using categorized forms of metabolites whereas sensitivity analyses were performed on continuous forms of phthalate metabolites with continuous and categorized forms of sex hormones. The cutoffs were established for androgen markers according to age groups. Since most studies consider age 50-51 years as the reference category for menopause, women with age≥ 50 years were considered postmenopausal and women with age < 50 years were considered premenopausal. Data-driven clustering among considered metabolites of phthalates and related standardized coefficients were used to create a composite score for each group. Furthermore, the grouping of phthalate metabolites was also done based on the molecular weight of metabolites or phthalate compounds. After obtaining grouping among metabolites and the composite exposure score for each group, the multivariable associations of log-transformed composite scores with TT, SHBG, obesity, and MS were examined using a survey weight-adjusted linear or Poisson regression

© 2022 Dubey P et al. *JAMA Network Open.*

analyses. The log transformation was made to improve the normality of continuous skewed phthalate metabolites and sex hormones data. The results were summarized using relative risk (RR) or regression coefficient (RC) along with a 95% confidence interval (CI) and p-value. In view of the analysis of multiple related outcomes, results from primary analysis were considered statistically significant at a 1% level of significance after adjusting for multiple comparisons.

© 2022 Dubey P et al. *JAMA Network Open.*

eAppendix. Supplemental Results

In the adjusted analyses of combined phthalate metabolites, the composite score of group 4 was positively associated with TT concentrations among postmenopausal women whereas other HMW was inversely associated with TT concentrations among premenopausal women (eTable 5). However, composite scores of group 2 (RC, -0.08 [95%CI, -0.12,-0.04]) or DNP-HMW score (RC, -0.06 [95%CI, -0.10,-0.03]) and group 4 (RC,-0.05 [95%CI, -0.08,-0.03]) or other HMW score (RC,-0.06 [95%CI, -0.10,-0.02]) were associated with lower SHBG concentrations to a greater extent among premenopausal women than among postmenopausal women (eTable 5). A combined score of all HMW metabolites was significantly associated with lower SHBG concentrations among premenopausal (RC,-0.10 [95%CI, -0.16, -0.05]) and postmenopausal (RC, -0.07 [95%CI,-0.13, -0.01]) women (eTable 5). However, obesity was positively associated with composite scores of group 2 (RR, 1.08 [95%CI, 1.02, 1.14]) and group 3 (RR, 1.09 [95%CI, 1.01, 1.17]) which were more pronounced among postmenopausal women than among premenopausal women. Composite scores of all HMW metabolites (RR, 1.12 [95%CI, 1.03, 1.23]) and other HMW metabolites (RR, 1.18 [95%CI, 1.05, 1.33]) were positively associated with obesity among premenopausal women while DNP related HMW metabolites (RR, 1.10 [95%CI, 1.00, 1.22]) was associated with obesity among postmenopausal women (eTable 6).

© 2022 Dubey P et al. *JAMA Network Open.*

eTable 1. Distribution of Exposure to Phthalate Metabolites

Phthalate metabolites	5th Percentile	25th Percentile	50th Percentile	75th Percentile	95th Percentile	T1 (range)	T2 (range)	T3 (range)
MCNP	0.3	0.9	1.8	3.7	13.0	0.1-1.2	1.3-2.8	2.8-876.4
MCOP	1.3	4.2	9.9	28.6	147.4	0.2-6.1	6.2-20.7	20.7-1019.8
MECPP	1.6	4.2	8.3	16.8	41.1	0.3-6.3	6.4-14.6	14.6-1421.9
MBP	1.1	4.3	9.8	18.4	48.6	0.3-6.8	6.9-16.5	16.6-1239.4
MCPP	0.3	0.5	1.2	2.9	12.5	0.3-0.7	0.8-2.0	2.0-449.5
MEP	3.6	12.9	33.6	97.4	521.5	0.9-21.4	21.5-75.2	75.2-22 983
MEHHP	0.9	2.6	5.2	10.7	27.8	0.3-3.8	3.9-9.3	9.3-1029.7
MEHP	0.6	0.6	0.9	2.1	6.2	0.6-0.6	0.6-1.7	1.7-152.3
MiBP	0.9	3.3	7.3	15.1	43.1	0.6-5.2	5.3-13.2	13.2-555.2
MNP	0.6	0.6	0.6	1.1	8.2	0.6-0.6	0.6-0.6	0.7-875.4
MEOHP	0.6	1.7	3.5	7.1	17.6	0.1-2.5	2.6-6.2	6.2-585.2
MBzP	0.4	1.5	3.8	9.1	34.0	0.2-2.2	2.3-6.8	6.8-340.1
MHNCH	0.3	0.3	0.3	0.7	3.3	0.3-0.3	0.3-0.5	0.5-151.7

Abbreviations: MBP, mono-n-butyl phthalate (ng/mL); MBzP, mono-benzyl phthalate (ng/mL); MCNP, mono(carboxynonyl) phthalate (ng/mL); MCOP, mono(carboxyoctyl) phthalate (ng/mL); MCPP, mono-(3-carboxypropyl) phthalate; MECPP, mono-2-ethyl-5-carboxypentyl phthalate; MEHHP, mono-(2-ethyl-5-hydroxyhexyl) phthalate; MEHP, mono-(2-ethyl)-hexyl phthalate (ng/mL); MEOHP, mono-(2-ethyl-5-oxohexyl) phthalate; MEP, mono-ethyl phthalate (ng/mL); MHNCH, cyclohexane 1, 2-dicarboxylic acid monohydroxy isononyl ester (ng/mL); MiBP, mono-isobutyl phthalate; MNP, mono-isononyl phthalate (ng/mL); T1, first tertile; T2, second tertile; T3, third tertile.

© 2022 Dubey P et al. *JAMA Network Open.*

eTable 2. Cluster Analysis of Metabolites of Phthalates

Group*	Metabolites	Weight/ phthalates	SRC	R2 with own Cluster	R2 with next Closest	1−R2	Variation explained by clusters	Proportion explained by clusters
**Group 1**	MCNP	HMW/DDP	0.17	0.46	0.08	0.59	4.48	0.34
	MECPP	HMW/ DEHP	0.23	0.91	0.10	0.10		
	MEHHP	HMW/ DEHP	0.24	0.96	0.08	0.05		
	MEHP	HMW/ DEHP	0.22	0.78	0.13	0.25		
	MEOHP	HMW/ DEHP	0.24	0.95	0.09	0.06		
**Group 2**	MCOP	HMW/ DNP	0.47	0.70	0.10	0.34	5.95	0.47
	MCPP	HMW/DiNOP	0.43	0.58	0.08	0.46		
	MNP	HMW/ DNP	0.40	0.50	0.03	0.52		
**Group 3**	MBP	LMW/DBP	0.47	0.54	0.02	0.46	7.39	0.57
	MEP	LMW/DEP	0.20	0.09	0.00	0.91		
	MiBP	LMW/DiBP	0.45	0.48	0.03	0.53		
	MBzP	HMW/BzBP	0.42	0.44	0.01	0.57		
**Group 4**	MHNCH	HMW/ DINCH	1.00	1.00	0.00	0.00	8.39	0.65

Abbreviations: SRC, standardized regression coefficient; MCNP, mono(carboxynonyl) phthalate (ng/mL); MCOP, mono (carboxyoctyl) phthalate(ng/mL); MECPP, mono-2-ethyl-5-carboxypentyl phthalate; MBP, mono-n-butyl phthalate (ng/mL); MCPP, mono-(3-carboxypropyl) phthalate; MEP, mono-ethyl phthalate (ng/mL); MEHHP, mono-(2-ethyl-5-hydroxyhexyl) phthalate; MEHP, mono-(2-ethyl)-hexyl phthalate (ng/mL); MiBP, mono-isobutyl phthalate; MNP, mono-isononyl phthalate (ng/mL); MEOHP, mono-(2-ethyl-5-oxohexyl) phthalate; MBzP, mono-benzyl phthalate (ng/mL); MHNCH, cyclohexane 1, 2-dicarboxylic acid monohydroxy isononyl ester (ng/mL); HMW, high molecular weight; LMW, low molecular weight; DDP, Di-isodecyl phthalate; DEHP, Di(2-ethylhexyl) phthalate; DNP, Di-isononyl phthalate; DiNOP, Di-n-octyl phthalate; DBP, Di-n-butyl phthalate; DEP, Di-ethyl phthalate; DiBP, Di-isobutyl phthalate; BzBP, benzylbutyl phthalate; DINCH, 1,2-Cyclohexane dicarboxylic acid, diisononyl ester; R2, coefficient of variation.

*Group 1 included 4 metabolites of DEHP and 1 metabolite of DDP and was represented by high molecular weight (HMW) with MCNP, MECPP, MEHHP, MEOHP, and MEHP. Group 2 was represented by 3 HMW metabolites of DNP and DiNOP. Group 3 included 3 low molecular weight (LMW) metabolites and 1 HMW metabolite and was represented by MBP, MiBP, and MBzP. Group 4 included HMW MHNCH, a metabolite of DINCH.

© 2022 Dubey P et al. *JAMA Network Open.*

eTable 3. Adjusted Association of Each Phthalate Metabolite With TT and SHBG Concentrations

	TT Concentration (N=1999)		SHBG Concentration (N=1862)	
Factor	RC* (95% CI)	p-value	RC* (95% CI)	p-value
MCNP				
T1				
T2	-0.067 (-0.176,0.043)	0.224	-0.114 (-0.218,-0.011)	0.031
T3	-0.035 (-0.128,0.059)	0.452	-0.185 (-0.268,-0.102)	<0.001
MCOP				
T1				
T2	-0.116 (-0.217,-0.016)	0.025	-0.068 (-0.142,0.006)	0.069
T3	-0.076 (-0.186,0.035)	0.173	-0.194 (-0.273,-0.114)	<0.001
MECPP				
T1				
T2	-0.023 (-0.127,0.081)	0.658	-0.063 (-0.132,0.006)	0.073
T3	0.023 (-0.07,0.115)	0.623	-0.116 (-0.176,-0.055)	<0.001
MBP				
T1				
T2	-0.043 (-0.139,0.052)	0.365	-0.028 (-0.118,0.063)	0.539
T3	0.025 (-0.076,0.126)	0.618	-0.052 (-0.147,0.042)	0.267
MCPP				
T1				
T2	-0.096 (-0.213,0.021)	0.104	-0.113 (-0.181,-0.044)	0.002
T3	-0.061 (-0.160,0.040)	0.206	-0.169 (-0.260,-0.080)	0.001
MEP				
T1				
T2	0.080 (-0.043,0.202)	0.196	-0.128 (-0.222,-0.033)	0.010
T3	0.028 (-0.082,0.139)	0.605	-0.054 (-0.167,0.058)	0.332
MEHHP				
T1				
T2	-0.045 (-0.153,0.063)	0.403	-0.047 (-0.150,0.056)	0.359
T3	-0.005 (-0.095,0.085)	0.911	-0.095 (-0.161,-0.029)	0.007
MEHP				
T1				
T2	0.010 (-0.064,0.083)	0.792	0.041 (-0.048,0.130)	0.358
T3	-0.009 (-0.105,0.086)	0.846	0.027 (-0.052,0.106)	0.496
MiBP				
T1				
T2	-0.014 (-0.137,0.110)	0.822	-0.083 (-0.155,-0.010)	0.026

© 2022 Dubey P et al. *JAMA Network Open.*

T3	-0.002 (-0.098,0.095)	0.972	-0.134 (-0.229,-0.038)	0.008
MNP				
T1/T2				
T3	-0.024 (-0.105,0.057)	0.551	-0.105 (-0.193,-0.018)	0.020
MEOHP				
T1				
T2	-0.016 (-0.116,0.084)	0.746	-0.055 (-0.152,0.041)	0.251
T3	0.006 (-0.087,0.099)	0.893	-0.054 (-0.121,0.013)	0.112
MBzP				
T1				
T2	-0.046 (-0.140,0.048)	0.324	-0.061 (-0.147,0.025)	0.158
T3	-0.005 (-0.093,0.082)	0.904	-0.075 (-0.174,0.024)	0.132
MHNCH				
T1				
T2	0.09 (-0.087,0.268)	0.307	-0.001 (-0.122,0.119)	0.984
T3	0.019 (-0.056,0.094)	0.606	-0.093 (-0.165,-0.021)	0.013

Abbreviations: MCNP, mono(carboxynonyl) phthalate (ng/mL); MCOP, mono (carboxyoctyl) phthalate(ng/mL); MECPP, mono-2-ethyl-5-carboxypentyl phthalate; MBP, mono-n-butyl phthalate (ng/mL); MCPP, mono-(3-carboxypropyl) phthalate; MEP, mono-ethyl phthalate (ng/mL); MEHHP, mono-(2-ethyl-5-hydroxyhexyl) phthalate; MEHP, mono-(2-ethyl)-hexyl phthalate (ng/mL); MiBP, mono-isobutyl phthalate; MNP, mono-isononyl phthalate (ng/mL); MEOHP, mono-(2-ethyl-5-oxohexyl) phthalate; MBzP, mono-benzyl phthalate (ng/mL); MHNCH, cyclohexane 1, 2-dicarboxylic acid monohydroxy isononyl ester (ng/mL); T1, first tertile; T2, second tertile; T3, third tertile; TT, total testosterone; SHBG, sex hormone-binding globulin; RC, regression coefficient; CI, confidence interval.
*All analyses adjusted for age, race and ethnicity, income, marital status, birth country, educational level, smoking status, alcohol use status, physical activity, and creatinine level.

© 2022 Dubey P et al. *JAMA Network Open.*

eTable 4. Adjusted Association of Each Phthalate Metabolite Concentration With TT and SHBG Concentrations

	Overall		Premenopausal		Postmenopausal	
	**RC* (95% CI)**	**p-value**	**RC* (95% CI)**	**p-value**	**RC* (95% CI)**	**p-value**
**TT concentration**						
MCNP	0.001 (-0.030,0.031)	0.969	-0.005 (-0.034,0.025)	0.758	0.006 (-0.054,0.066)	0.845
MCOP	-0.018 (-0.049,0.013)	0.246	-0.010 (-0.036,0.016)	0.427	-0.027 (-0.087,0.033)	0.366
MECPP	-0.001 (-0.035,0.033)	0.942	0.001 (-0.033,0.035)	0.940	-0.006 (-0.066,0.055)	0.852
MBP	-0.006 (-0.045,0.032)	0.734	0.005 (-0.019,0.029)	0.677	-0.023 (-0.098,0.051)	0.524
MCPP	-0.022 (-0.059,0.015)	0.228	-0.008 (-0.040,0.023)	0.594	-0.043 (-0.118,0.032)	0.253
MEP	-0.004 (-0.036,0.028)	0.807	0.003 (-0.026,0.031)	0.851	-0.008 (-0.064,0.049)	0.785
MEHHP	-0.019 (-0.053,0.015)	0.266	0.001 (-0.031,0.033)	0.938	-0.049 (-0.115,0.018)	0.144
MEHP	-0.005 (-0.045,0.036)	0.820	0.019 (-0.024,0.062)	0.369	-0.054 (-0.120,0.013)	0.109
MiBP	-0.008 (-0.042,0.026)	0.625	0.012 (-0.009,0.034)	0.241	-0.038 (-0.097,0.021)	0.201
MNP	-0.023 (-0.056,0.010)	0.171	-0.012 (-0.047,0.023)	0.483	-0.043 (-0.150,0.063)	0.413
MEOHP	-0.013 (-0.051,0.026)	0.501	0.006 (-0.026,0.038)	0.694	-0.043 (-0.118,0.032)	0.255
MBzP	-0.006 (-0.029,0.016)	0.577	-0.014 (-0.038,0.010)	0.234	-0.004 (-0.043,0.035)	0.844
MHNCH	0.010 (-0.024,0.045)	0.542	-0.010 (-0.041,0.020)	0.492	0.062 (0.009,0.114)	0.023
**SHBG concentration**						
MCNP	-0.066 (-0.099,-0.032)	<0.001	-0.077 (-0.117,-0.038)	<0.001	-0.061 (-0.114,-0.009)	0.024
MCOP	-0.064 (-0.089,-0.038)	<0.001	-0.068 (-0.099,-0.037)	<0.001	-0.058 (-0.098,-0.017)	0.007
MECPP	-0.041 (-0.074,-0.008)	0.018	-0.059 (-0.100,-0.019)	0.006	-0.025 (-0.072,0.022)	0.286
MBP	-0.030 (-0.063,0.003)	0.075	-0.023 (-0.066,0.020)	0.277	-0.037 (-0.077,0.003)	0.071
MCPP	-0.058 (-0.091,-0.026)	0.001	-0.067 (-0.104,-0.031)	0.001	-0.046 (-0.092,0.00)	0.048
MEP	-0.007 (-0.033,0.019)	0.567	0.00 (-0.039,0.040)	0.991	-0.017 (-0.050,0.016)	0.308
MEHHP	-0.034 (-0.065,-0.002)	0.037	-0.038 (-0.079,0.003)	0.069	-0.031 (-0.069,0.007)	0.106
MEHP	0.015 (-0.022,0.051)	0.420	-0.006 (-0.05,0.037)	0.765	0.051 (-0.014,0.116)	0.123
MiBP	-0.047 (-0.080,-0.014)	0.007	-0.048 (-0.099,0.003)	0.064	-0.048 (-0.089,-0.007)	0.022
MNP	-0.048 (-0.092,-0.004)	0.033	-0.045 (-0.103,0.014)	0.130	-0.040 (-0.117,0.036)	0.292
MEOHP	-0.015 (-0.046,0.016)	0.331	-0.018 (-0.058,0.022)	0.375	-0.021 (-0.058,0.015)	0.242
MBzP	-0.034 (-0.058,-0.011)	0.005	-0.037 (-0.071,-0.003)	0.033	-0.034 (-0.076,0.007)	0.099
MHNCH	-0.058 (-0.088,-0.027)	<0.001	-0.070 (-0.114,-0.026)	0.003	-0.045 (-0.092,0.001)	0.057

Abbreviations: MCNP, mono(carboxynonyl) phthalate (ng/mL); MCOP, mono (carboxyoctyl) phthalate(ng/mL); MECPP, mono-2-ethyl-5-carboxypentyl phthalate; MBP, mono-n-butyl phthalate (ng/mL); MCPP, mono-(3-carboxypropyl) phthalate; MEP, mono-ethyl phthalate (ng/mL); MEHHP, mono-(2-ethyl-5-hydroxyhexyl) phthalate; MEHP, mono-(2-ethyl)-hexyl phthalate (ng/mL); MiBP, mono-isobutyl phthalate; MNP, mono-isononyl phthalate (ng/mL); MEOHP, mono-(2-ethyl-5-oxohexyl) phthalate; MBzP, mono-benzyl phthalate (ng/mL); MHNCH, cyclohexane 1, 2-dicarboxylic acid monohydroxy isononyl ester (ng/mL); TT, total testosterone; SHBG, sex hormone-binding globulin; RC, regression coefficient; CI, confidence interval.

© 2022 Dubey P et al. *JAMA Network Open.*

[*]Phthalate metabolites, SHBG and TT were log-transformed in the analyses and all analyses adjusted for age, race and ethnicity, income, marital status, birth country, educational level, smoking status, alcohol use status, physical activity, and creatinine level.

© 2022 Dubey P et al. *JAMA Network Open.*

eTable 5. Adjusted Association of Each Phthalate Metabolite Concentration With Obesity and Metabolic Syndrome

	**Overall**		**Premenopausal**		**Postmenopausal**	
	**RR* (95% CI)**	**p-value**	**RR* (95% CI)**	**p-value**	**RR* (95% CI)**	**p-value**
**Obesity**						
MCNP	1.098 (1.036,1.164)	0.003	1.079 (0.995,1.171)	0.065	1.142 (1.051,1.240)	0.003
MCOP	1.102 (1.053,1.153)	<0.001	1.088 (1.024,1.155)	0.007	1.126 (1.050,1.207)	0.002
MECPP	1.126 (1.053,1.205)	0.001	1.128 (1.026,1.241)	0.014	1.137 (1.030,1.256)	0.013
MBP	1.138 (1.066,1.216)	<0.001	1.165 (1.063,1.277)	0.002	1.115 (1.022,1.217)	0.017
MCPP	1.081 (1.028,1.136)	0.004	1.056 (0.985,1.133)	0.122	1.116 (1.033,1.206)	0.007
MEP	1.064 (1.009,1.123)	0.023	1.044 (0.973,1.120)	0.219	1.103 (1.022,1.189)	0.013
MEHHP	1.114 (1.032,1.202)	0.007	1.112 (1.013,1.220)	0.026	1.118 (1.006,1.242)	0.039
MEHP	0.972 (0.904,1.045)	0.426	0.990 (0.901,1.087)	0.823	0.931 (0.804,1.079)	0.331
MiBP	1.141 (1.062,1.226)	0.001	1.158 (1.046,1.283)	0.006	1.125 (1.024,1.235)	0.016
MNP	1.029 (0.946,1.120)	0.494	0.996 (0.895,1.108)	0.933	1.097 (0.960,1.255)	0.166
MEOHP	1.117 (1.033,1.207)	0.007	1.123 (1.025,1.230)	0.014	1.119 (1.002,1.250)	0.047
MBzP	1.128 (1.064,1.196)	<0.001	1.182 (1.103,1.267)	<0.001	1.073 (0.988,1.164)	0.090
MHNCH	1.072 (1.010,1.136)	0.023	1.087 (1.007,1.174)	0.035	1.049 (0.948,1.162)	0.343
**Metabolic syndrome**						
MCNP	1.018 (0.964,1.074)	0.511	1.070 (0.999,1.146)	0.052	0.993 (0.928,1.063)	0.830
MCOP	1.023 (0.984,1.063)	0.239	1.015 (0.953,1.082)	0.632	1.034 (0.986,1.083)	0.161
MECPP	1.054 (0.985,1.128)	0.121	1.097 (1.007,1.196)	0.035	1.029 (0.956,1.108)	0.439
MBP	1.053 (0.99,1.121)	0.097	1.063 (0.957,1.180)	0.246	1.052 (0.987,1.122)	0.115
MCPP	1.00 (0.955,1.048)	0.996	1.003 (0.943,1.066)	0.918	1.001 (0.943,1.063)	0.967
MEP	1.018 (0.975,1.064)	0.404	1.024 (0.973,1.077)	0.351	1.025 (0.969,1.085)	0.380
MEHHP	1.081 (1.010,1.157)	0.025	1.107 (1.017,1.205)	0.020	1.061 (0.986,1.141)	0.108
MEHP	0.980 (0.911,1.053)	0.566	1.031 (0.937,1.135)	0.519	0.925 (0.842,1.017)	0.104
MiBP	1.054 (0.980,1.132)	0.149	1.043 (0.950,1.146)	0.361	1.067 (0.985,1.155)	0.109
MNP	0.997 (0.923,1.076)	0.930	0.997 (0.901,1.102)	0.950	1.003 (0.912,1.104)	0.947
MEOHP	1.061 (0.99,1.137)	0.091	1.107 (1.011,1.211)	0.029	1.031 (0.959,1.107)	0.401
MBzP	1.076 (1.021,1.133)	0.008	1.076 (0.987,1.172)	0.092	1.090 (1.035,1.147)	0.002
MHNCH	1.027 (0.979,1.078)	0.265	1.071 (1.015,1.130)	0.014	0.980 (0.908,1.058)	0.595

Abbreviations: MCNP, mono(carboxynonyl) phthalate (ng/mL); MCOP, mono (carboxyoctyl) phthalate(ng/mL); MECPP, mono-2-ethyl-5-carboxypentyl phthalate; MBP, mono-n-butyl phthalate (ng/mL); MCPP, mono-(3-carboxypropyl) phthalate; MEP, mono-ethyl phthalate (ng/mL); MEHHP, mono-(2-ethyl-5-hydroxyhexyl) phthalate; MEHP, mono-(2-ethyl)-hexyl phthalate (ng/mL); MiBP, mono-isobutyl phthalate; MNP, mono-isononyl phthalate (ng/mL); MEOHP, mono-(2-ethyl-5-oxohexyl) phthalate; MBzP, mono-benzyl phthalate (ng/mL); MHNCH, cyclohexane 1, 2-dicarboxylic acid monohydroxy isononyl ester (ng/mL); TT, total testosterone; SHBG, sex hormone-binding globulin; RR, relative risk; CI, confidence interval.

*Phthalate metabolites were log-transformed in the analyses and all analyses adjusted for age, race and ethnicity, income, marital status, birth country, educational level, smoking status, alcohol use status, physical activity, and creatinine level. .

© 2022 Dubey P et al. *JAMA Network Open.*

eTable 6. Adjusted Associations of Composite Scores of Phthalate Metabolites With TT and SHBG Concentrations

	Overall		Premenopausal		Postmenopausal	
	**RC* (95% CI)**	**p-value**	**RC* (95% CI)**	**p-value**	**RC* (95% CI)**	**p-value**
**TT concentration**						
**Data-driven groups**						
Composite score 1[a]	0.010 (-0.044,0.064)	0.707	0.019 (-0.035,0.072)	0.481	-0.010 (-0.118,0.098)	0.849
Composite score 2[b]	-0.022 (-0.061,0.016)	0.243	-0.017 (-0.049,0.015)	0.286	-0.029 (-0.112,0.053)	0.473
Composite score 3[c]	-0.007 (-0.053,0.039)	0.759	0.004 (-0.041,0.050)	0.844	-0.015 (-0.089,0.059)	0.675
Composite score 4[d]	0.013 (-0.019,0.045)	0.418	-0.014 (-0.046,0.018)	0.383	0.072 (0.028,0.117)	0.002
**Molecular weight-based groups**						
HMW score[e]	-0.018 (-0.057,0.022)	0.368	-0.015 (-0.055,0.026)	0.456	-0.026 (-0.108,0.056)	0.523
LMW score[f]	-0.0003 (-0.038,0.037)	0.987	0.011 (-0.026,0.048)	0.553	-0.008 (-0.069,0.052)	0.779
**Compounds based groups**						
DEHP-HMW score[g]	0.009 (-0.043,0.062)	0.720	0.031 (-0.023,0.086)	0.251	-0.024 (-0.128,0.081)	0.648
DNP-HMW score[h]	-0.022 (-0.059,0.015)	0.232	-0.008 (-0.041,0.024)	0.61	-0.036 (-0.116,0.044)	0.361
LMW score[i]	-0.003 (-0.044,0.039)	0.888	0.012 (-0.029,0.054)	0.552	-0.009 (-0.076,0.058)	0.777
Other-HMW score[j]	-0.001 (-0.031,0.030)	0.973	-0.040 (-0.079,-0.000)	0.049	0.036 (-0.020,0.092)	0.201
**SHBG concentration**						
Composite score 1[a]	0.024 (-0.029,0.078)	0.361	0.007 (-0.060,0.074)	0.831	0.036 (-0.024,0.095)	0.228
Composite score 2[b]	-0.076 (-0.115,-0.038)	<0.001	-0.079 (-0.122,-0.036)	0.001	-0.071 (-0.13,-0.012)	0.021
Composite score 3[c]	0.009 (-0.031,0.049)	0.654	0.033 (-0.028,0.093)	0.279	-0.016 (-0.067,0.035)	0.531
Composite score 4[d]	-0.054 (-0.083,-0.026)	<0.001	-0.069 (-0.112,-0.027)	0.002	-0.038 (-0.084,0.007)	0.095
**Molecular weight-based groups**						
HMW score[e]	-0.085 (-0.126,-0.045)	<0.001	-0.103 (-0.155, -0.052)	<0.001	-0.067 (-0.125,-0.011)	0.022
LMW score[f]	0.017 (-0.018,0.052)	0.318	0.034 (-0.017,0.085)	0.186	-0.001 (-0.044,0.042)	0.964
**Compounds based groups**						
DEHP-HMW score[g]	0.042 (-0.009,0.093)	0.102	0.027 (-0.038,0.091)	0.406	0.061 (-0.004,0.126)	0.066
DNP-HMW score[h]	-0.064 (-0.104,-0.025)	0.002	-0.062 (-0.109,-0.015)	0.012	-0.063 (-0.125,0.000)	0.049
LMW score[i]	0.013 (-0.024,0.049)	0.482	0.035 (-0.021,0.091)	0.214	-0.009 (-0.054,0.035)	0.670
Other-HMW score[j]	-0.058 (-0.095,-0.020)	0.004	-0.074 (-0.13,-0.017)	0.012	-0.054 (-0.117,0.010)	0.096

Abbreviations: TT, total testosterone; SHBG, sex hormone-binding globulin; RC, regression coefficient; CI, confidence interval.

[a] Composite score1 includes mono(carboxynonyl) phthalate (ng/mL), mono-2-ethyl-5-carboxypentyl phthalate, mono-(2-ethyl-5-hydroxyhexyl) phthalate, mono-(2-ethyl)-hexyl phthalate (ng/mL) and mono-(2-ethyl-5-oxohexyl) phthalate.

[b] Composite score 2 includes mono (carboxyoctyl) phthalate(ng/mL), mono-(3-carboxypropyl) phthalate and mono-isononyl phthalate (ng/mL).

© 2022 Dubey P et al. *JAMA Network Open.*

[c] Composite score 3 includes mono-n-butyl phthalate (ng/mL), mono-ethyl phthalate (ng/mL), mono-isobutyl phthalate and mono-benzyl phthalate (ng/mL).
[d] Composite score 4 includes only cyclohexane 1, 2-dicarboxylic acid monohydroxy isononyl ester (ng/mL).
[e]High molecular weight (HMW) score is the sum of all HMW phthalate metabolite concentrations including MCNP, MECPP, MEHHP, MEHP, MEOHP, MCOP, MCPP, MNP, MBzP, and MHNCH.
[f]low molecular weight (LMW) score is the sum of all LMW phthalate metabolite concentrations including MBP, MEP, and MiBP.
[g]DEHP-HMW score is the total concentration of di(2-ethylhexyl) phthalate metabolites (DEHP) including MECPP, MEHHP, MEHP, and MEOHP.
[h]DNP-HMW score is the total concentration of di-isononyl phthalate metabolites (DNP) including MCOP and MNP and MCPP metabolite of di-n-octyl phthalate (DiNOP).
[i]LMW score is the total concentration of LMW phthalate metabolites MBP, MEP, and MiBP.
[j]other-HMW score is the total concentration of MCNP, MBzP, and MHNCH.

*Composite scores, SHBG and TT were log-transformed in the analyses and all analyses adjusted for age, race and ethnicity, income, marital status, birth country, educational level, smoking status, alcohol use status, physical activity, and creatinine level.

© 2022 Dubey P et al. *JAMA Network Open.*

eTable 7. Adjusted Associations of Composite Scores of Phthalate Metabolites With Obesity and Metabolic Syndrome

	**Overall**		**Premenopausal**		**Postmenopausal**	
	**RR* (95% CI)**	**p-value**	**RR* (95% CI)**	**p-value**	**RR* (95% CI)**	**p-value**
**Obesity**						
**Data-driven groups**						
Composite score 1[a]	1.001 (0.917,1.092)	0.984	1.015 (0.912,1.128)	0.783	0.972 (0.833,1.134)	0.712
Composite score 2[b]	1.075 (1.017,1.136)	0.012	1.052 (0.978,1.131)	0.163	1.111 (1.005,1.228)	0.040
Composite score 3[c]	1.087 (1.009,1.171)	0.030	1.078 (0.987,1.178)	0.091	1.114 (0.991,1.252)	0.069
Composite score 4[d]	1.043 (0.976,1.115)	0.203	1.060 (0.979,1.147)	0.146	1.014 (0.912,1.128)	0.784
**Molecular weight-based groups**						
HMW score[e]	1.122 (1.041,1.210)	0.004	1.122 (1.028,1.225)	0.012	1.120 (0.995,1.262)	0.061
LMW score[f]	1.052 (0.983,1.127)	0.138	1.036 (0.957,1.122)	0.366	1.085 (0.976,1.205)	0.125
**Compounds based groups**						
DEHP-HMW score[g]	0.986 (0.881,1.103)	0.797	0.982 (0.862,1.119)	0.784	0.978 (0.827,1.157)	0.791
DNP-HMW score[h]	1.054 (0.987,1.124)	0.112	1.018 (0.938,1.105)	0.658	1.103 (1.000,1.218)	0.051
LMW score[i]	1.045 (0.974,1.121)	0.208	1.011 (0.936,1.092)	0.766	1.093 (0.981,1.218)	0.102
Other-HMW score[j]	1.105 (0.985,1.239)	0.087	1.182 (1.052,1.327)	0.006	1.025 (0.888,1.183)	0.730
**Metabolic syndrome**						
**Data-driven groups**						
Composite score 1[a]	1.055 (0.955,1.165)	0.282	1.122 (0.980,1.284)	0.094	0.988 (0.880,1.109)	0.834
Composite score 2[b]	0.990 (0.941,1.041)	0.678	0.967 (0.873,1.072)	0.512	1.019 (0.958,1.085)	0.536
Composite score 3[c]	1.024 (0.956,1.096)	0.485	0.994 (0.908,1.087)	0.890	1.062 (0.977,1.155)	0.153
Composite score 4[d]	1.011 (0.957,1.067)	0.697	1.049 (0.989,1.113)	0.105	0.968 (0.890,1.053)	0.439
**Molecular weight-based groups**						
HMW score[e]	1.041 (0.965,1.122)	0.289	1.067 (0.973,1.171)	0.163	1.026 (0.938,1.122)	0.563
LMW score[f]	1.019 (0.962,1.080)	0.507	1.012 (0.943,1.087)	0.725	1.034 (0.961,1.113)	0.356
**Compounds based groups**						
DEHP-HMW score[g]	1.043 (0.965,1.128)	0.273	1.083 (0.961,1.22)	0.184	0.988 (0.888,1.099)	0.820
DNP-HMW score[h]	0.983 (0.934,1.035)	0.511	0.952 (0.857,1.057)	0.341	1.010 (0.946,1.078)	0.757
LMW score[i]	1.009 (0.951,1.069)	0.767	0.987 (0.915,1.063)	0.717	1.033 (0.958,1.113)	0.389
Other-HMW score[j]	1.042 (0.977,1.110)	0.202	1.092 (0.983,1.214)	0.098	1.037 (0.943,1.139)	0.442

Abbreviations: RR, relative risk; CI, confidence interval.

© 2022 Dubey P et al. *JAMA Network Open.*

[a] Composite score1 includes mono(carboxynonyl) phthalate (ng/mL)-MCNP, mono-2-ethyl-5-carboxypentyl phthalate-MECPP, mono-(2-ethyl-5-hydroxyhexyl) phthalate-MEHHP, mono-(2-ethyl)-hexyl phthalate (ng/mL)-MEHP and mono-(2-ethyl-5-oxohexyl) phthalate-MEOHP.
[b] Composite score 2 includes mono (carboxyoctyl) phthalate(ng/mL)-MCOP, mono-(3-carboxypropyl) phthalate(ng/mL)-MCPP and mono-isononyl phthalate (ng/mL)-MNP.
[c] Composite score 3 includes mono-n-butyl phthalate (ng/mL)-MBP, mono-ethyl phthalate (ng/mL)-MEP, mono-isobutyl phthalate(ng/mL)-MiBP and mono-benzyl phthalate (ng/mL)-MBzP.
[d] Composite score 4 includes only cyclohexane 1, 2-dicarboxylic acid monohydroxy isononyl ester (ng/mL)-MHNCH.
[e]High molecular weight (HMW) score is the sum of all HMW phthalate metabolite concentrations including MCNP, MECPP, MEHHP, MEHP, MEOHP, MCOP, MCPP, MNP, MBzP, and MHNCH.
[f]low molecular weight (LMW) score is the sum of all LMW phthalate metabolite concentrations including MBP, MEP, and MiBP.
[g]DEHP-HMW score is the total concentration of di(2-ethylhexyl) phthalate metabolites (DEHP) including MECPP, MEHHP, MEHP, and MEOHP.
[h]DNP-HMW score is the total concentration of di-isononyl phthalate metabolites (DNP) including MCOP and MNP and MCPP metabolite of di-n-octyl phthalate (DiNOP).
[i]LMW score is the total concentration of LMW phthalate metabolites MBP, MEP, and MiBP.
[j]other-HMW score is the total concentration of MCNP, MBzP, and MHNCH.

*All scores were log-transformed in the analyses and all analyses adjusted for age, race and ethnicity, income, marital status, birth country, educational level, smoking status, alcohol use status, physical activity, and creatinine level.

© 2022 Dubey P et al. *JAMA Network Open.*
